# How Close Are We to the Production of Milk in Alternative Systems? The Fat Perspective

**DOI:** 10.3390/foods14050809

**Published:** 2025-02-26

**Authors:** Roni Tadmor-Levi, Nurit Argov-Argaman

**Affiliations:** Department of Animal Sciences, RH Smith Faculty of Agriculture, Food and Environment, The Hebrew University of Jerusalem, Rehovot 7610001, Israel

**Keywords:** dairy, lipids, synthetic biology, sustainable foods, oleaginous microorganisms

## Abstract

The growing demand for sustainable food systems has led to significant advancements in developing alternatives to animal-derived products. Dairy products are an important dietary source of proteins and fats; however, their production raises environmental concerns, including greenhouse gas emissions, extensive land and water usage, and biodiversity loss. Therefore, there is a need to develop sustainable, scalable solutions that will enable the production of quality replacements for animal-based foods with reduced environmental impacts. Recognizing that replacing animal-based products from a single source is currently not feasible; there is a need for high-quality sources of ingredients that can be combined to mimic the holistic product. In recent years, plant-based dairy alternatives have gained traction; however, their inability to replicate the sensorial experience of real milk—attributed largely to the unique composition and structure of milk fat—remains a key limitation. Cow’s milk fat has distinctive characteristics, including a complex fatty acid profile, which is rich in short- and medium-chain saturated fatty acids with specific positional distribution. These characteristics of cow’s milk play a role in delivering the aroma, texture, and mouthfeel of dairy products. Recent efforts have focused on leveraging precision fermentation and cellular agriculture to mimic these properties. This review explores the unique lipid composition of ruminant milk, the biosynthesis of milk fats, and the challenges of replicating these features in non-mammalian systems. Emphasis is placed on short-chain fatty acids and chain-termination mechanisms in fatty acid synthesis. By integrating insights from diverse biological systems, we aim to contribute to a deeper understanding of the complex processes related to milk fat synthesis.

## 1. Introduction

Milk and dairy products are an important dietary source for proteins, fats, and vitamins. In recent years, the demand for animal product alternatives has significantly risen due to numerous reasons, such as animal welfare, environmental concerns, and consumers’ health concerns like milk allergies or intolerance. Environmental concerns are of utmost importance, as dairy farming has a significant ecological footprint. Intensive dairy farming contributes significantly to greenhouse gas emissions, primarily methane from gastric fermentation in the rumen [1]. Additionally, extensive land use leads to deforestation, and as forests are cleared for pasture, a significant loss of biodiversity is observed [2]. High water consumption required for both the cows and the production of their feed strains local water resources [3], while manure runoff contaminates waterways, leading to eutrophication and harming aquatic ecosystems [4]. Therefore, the food industry has been making immense efforts over the last decade to develop alternatives for animal-based food products, mainly through precision fermentation and cellular agriculture.

Oleaginous microorganisms of various types are emerging as a sustainable source of oils, offering a promising avenue for mimicking animal fat. Some of these organisms have advanced genetic engineering tools and can be genetically altered to synthesize desirable lipids. These are currently utilized mainly for the production of essential polyunsaturated fatty acids or saturated fatty acids for biodiesel [5] and are emerging now as a sustainable source for edible oils.

Current dairy alternatives available on the market are based on plants and plant oils, and are inferior to real milk in terms of taste, texture, mouthfeel, and technological properties. They are also more expensive and less reachable to broader audiences. In dairy alternatives, the major limitation up to date is mimicking the sensorial experience of consumption of real dairy products. Efforts to mimic milk using precision fermentation have been focused on milk proteins and not on milk fats. However, the sensorial experience of consumption of real dairy products is largely attributed to the unique composition of dairy fat. This experience is attributed to both the chemical composition and the unique molecular arrangement of milk fats, which are considered largely accountable for the flavor, aroma, and mouthfeel of real milk. Thus, further efforts should be made to produce lipids that can successfully replace milk fats in alternative products.

In this review, we will present the unique properties of dairy fat, layout the challenges to produce dairy like fats, and the advancements that have been made in recent years.

## 2. Lipid Composition in Ruminant’s Milk

Although the mechanisms for producing fats and oils are highly conserved throughout evolution from bacteria to higher eukaryotes, there is a great variability between organisms in lipid composition [6,7]. This includes differences in classes of lipids, fatty acid composition, and their positional distribution in storage lipids. Additionally, in higher organisms, different tissues have distinct fatty acid compositions even in the same organism. For example, milk fat produced by the mammary gland of cows is unique, differing both from milks of other mammals (mostly non-ruminants), as well as from other non-mammary bovine lipogenic tissues [8,9,10]. The fatty acid composition of milk is highly diverse, with over 400 different fatty acids differing in length, saturation levels, branching, etc. Most of the fatty acids in milk are saturated, with the main fatty acid being palmitic acid (C16:0). Additionally, short- and medium-chain fatty acids are found at a relatively high abundance in ruminants’ milk, with a relative abundance that can reach over 25% of total fatty acids [10]. While in liver and adipose tissues of cows certain amounts of myristic acid (C14:0) are typically found [11,12], the shorter chained fatty acids (C12:0 and shorter) are exclusively found in the mammary gland and not in any extra-mammary tissues. These and other fatty acids are not randomly esterified to the three positions of the glycerol backbone. In dairy fat, short-chain fatty acids are prominently esterified to the sn-3 position, while medium- and long-chain fatty acids are more variable in their positions, while still maintaining a preferential position for each type of fatty acid [13]. These chemical characteristics are responsible for the unique technological properties of ruminants’ milk (like solid fat content), and hence contribute to the rheological properties of dairy products with a distinguished texture, mouthfeel, and taste [14]. On top of that, when dairy fat is ingested, the digestion process starts in the mouth by a lingual lipase. This enzyme hydrolases the sn-3 position of the glycerol backbone of the triglyceride, releasing mainly short-chain volatile fatty acids, which contribute to the aroma and taste of dairy products [15]. Thus, both the occurrence as well as the specific position of short-chain fatty acids in dairy fat are responsible for the sensorial experience induced by the consumption of dairy fats and not by plant oils or other animal fat sources (meat).

## 3. Production of Bovine Milk Fat and *De Novo* Fatty Acid Synthesis

Approximately 98% milk fat is in the form of triglycerides, and the rest of the lipid mass is in the form of phospholipids, cholesterol, and cholesteryl esters. Milk lipids are produced from fatty acids that are either supplied from the blood system as preformed fatty acids or synthesized *de novo* in the mammary gland epithelial cells by fatty acids synthase (FAS, FASN) enzymatic complex. While long-chain fatty acids are absorbed from the blood stream, short- and medium-chain fatty acids are almost exclusively produced *de novo* in the mammary gland [8]. The mechanism of fatty acid *de novo* synthesis is highly conserved across organisms and is common to animals, plants, yeast, and bacteria [16]. Although the biochemical pathways and enzymes enabling the production of fatty acids are conserved across organisms, some structural differences occur between phyla. In vertebrates, including ruminants, the FASN complex consists of a homo-dimer, with each unit consisting of seven catalytic sites (type I FASN, [17]). In yeast, the seven catalytic sites are split into two separate proteins, which form a hetero-dodecamer complex [18]. In plants and bacteria, the seven catalytic sites are split into seven separate proteins (type II FAS, [19]).

**Figure 1:**
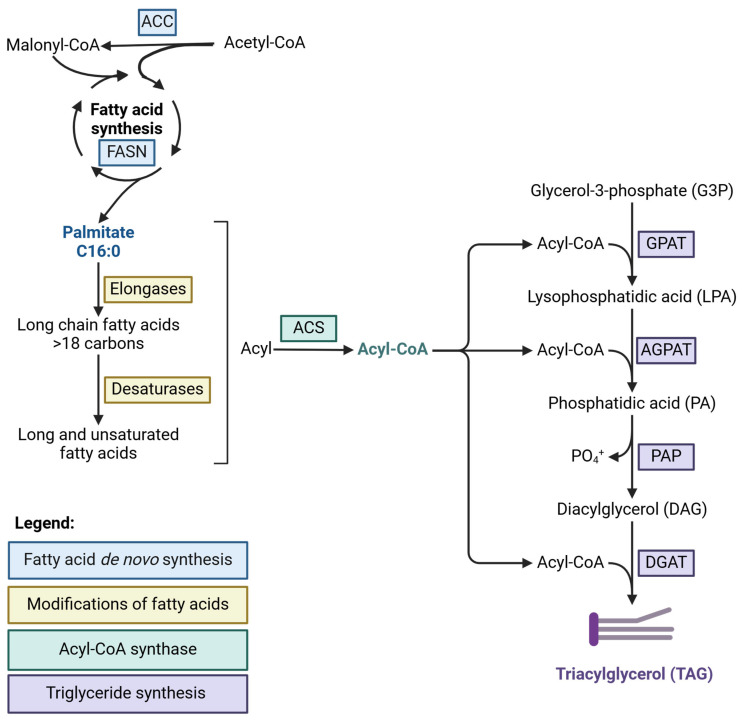
Simplified overview of fatty acid and triglyceride synthesis. The *de novo* fatty acid synthesis begins with the carboxylation of acetyl-CoA to malonyl-CoA by acetyl-CoA carboxylase (ACC). Fatty acid synthase (FASN) then catalyzes the sequential addition of two-carbon units from malonyl-CoA to a growing acyl chain, typically resulting in palmitate (C16:0). Palmitate can be further modified by elongases and desaturases to produce longer and/or unsaturated fatty acids. Acyl-CoA synthase (ACS) activates fatty acids by converting them to Acyl-CoA, which is then used for the synthesis of polar lipids or triglycerides. Triglyceride synthesis involves the sequential acylation of glycerol-3-phosphate (G3P) by glycerol-3-phosphate acyltransferase (GPAT), acylglycerol-3-phosphate *O*-acyltransferase (AGPAT), and diacylglycerol *O*-acyltransferase (DGAT), resulting in the formation of triacylglycerol (TAG). This figure was created with https://BioRender.com.

The milk lipid synthesis process is illustrated in Figure 1 and detailed herein. Synthesis of fatty acids is governed by FASN and initiated with the loading of acetyl-CoA to the FASN complex, followed by sequential elongation steps in which two carbons acquired from malonyl-CoA are added. The production of malonyl-CoA is governed mainly by acetyl-CoA carboxylase (ACC), which is considered a rate-limiting step in fatty acid synthesis. The sequential elongation process in all organisms is typically terminated when reaching a chain length of 16 carbons (C16:0, palmitate) with a release of fatty acids by a thioesterase [17,20,21]. Fatty acids can be subjected to further modifications of the addition of double bonds (de-saturation) or elongation catalyzed by desaturases or elongases, respectively [22]. The products of FASN or the elongation and desaturation steps are utilized for the production of complex lipids like polar or neutral lipids (i.e., phospholipids, sphingolipids, and triglycerides). Prior to their utilization in more complex lipids, fatty acids are activated by esterification to Coenzyme A (CoA). The majority (c.a. 98%) of fatty acids in milk fat are found in the form of triglycerides [8] with a molecular structure of three fatty acids esterified to a glycerol backbone. The triglyceride synthesis requires sequential steps of esterification of acyl-CoA to a glycerol backbone with specific fatty acid positions [6]. Each step in the triglyceride synthesis process is carried out by a gene family with several isomers [23]. Although there is some redundancy between family members, in bovine, a tissue-specific expression pattern for isomers in the triglyceride synthesis steps is observed [24].

In summary, the production of milk triglycerides involves a complex interplay of *de novo* fatty acid synthesis, modifications, and assembly. The intricate nature of these processes, involving multiple enzymes and regulatory steps, presents significant challenges for replicating milk fat production outside of the mammary gland context. Thus, unlike the direct gene-to-protein relationship, that facilitates animal protein production by precision fermentation, biosynthesis of lipids is more challenging to reproduce in other systems.

## 4. The Occurrence of Short-Chain Fatty Acids in Non-Ruminant Organisms

The way in which the mammary gland of cows and other ruminants produce their unique lipid composition, with a high abundance of short- and medium-chained fatty acids in specific positions, is largely unknown. We recently suggested that it is a result of a combination of three major properties: (i) a specialized acyl-transferase unit of the fatty acid synthase (FASN/FAS) complex, with an ability to load, and more importantly, release short-chain fatty acids esterified to a CoA (as suggested by [25]). (ii) An enzymatic environment that favors ligation of CoA to shorter acyl chains over ligation to long-chain fatty acids. Thus, production is shifted towards the production of shorter-chained fatty acids that are more available for utilization in triglycerides. (iii) Rapid downstream utilization of these fatty acids into triglycerides in a specific positional distribution facilitated by a unique expression pattern of triglyceride synthesis genes in the mammary gland [24].

Other organisms also produce, and some even accumulate, several types of short- and medium-chained fatty acids. Table 1 summarizes the types, sources, and storage forms of short- and medium-chain fatty acids in different non-ruminant organisms.

The production and storage of short- and medium-chain fatty acids vary significantly across different organisms (Table 1). The production of short-chain fatty acids (mainly C2–C4) by fermentation of dietary fibers occurs in the colon and cecum of monogastric animals [26] and the rumen of ruminants [27]. However, these fatty acids are released in their free form and are not accumulated in triglycerides like they are in dairy fat. Tropical plants, like coconut and palm, accumulate medium-chain fatty acids (mainly C8–C14) in their seeds as triglycerides [28,29]. Currently, these oils are widely used in the food industry. When used to replace dairy fat, it does not provide the sensorial experience produced by real dairy fats. Moreover, these sources of oil require depletion of natural resources and deforestation, which makes them highly unsustainable. Other warm climate plants like California bay (*Umbellularia californica*) or *Cuphea* spp. also produce and accumulate medium-chain fatty acids (mainly C8–C12) in their seeds [30,31]. However, these plants are not suitable for commercial oil production. In the animal kingdom, aquatic birds produce medium-chain fatty (mainly C12–C14) acids in the preen glands, which are utilized in the wax they secrete to waterproof their feathers [32]. Some non-ruminant herbivorous mammals, such as rodents [33] or camelids [34], produce medium-chain fatty acids in their mammary glands, which are secreted in milk as triglycerides.

Despite these sources of short- and medium-chain fatty acids in nature (Table 1), their composition and positional distribution on triglycerides differ significantly from that of dairy fat. Ruminant milk fat is characterized by even shorter-chain fatty acids (C4, C6) and a distinct positional distribution, resulting in distinguished technological properties. Furthermore, many of these alternative sources (e.g., rodent milk) are impractical for large-scale production. Nevertheless, the similarity in lipid biosynthesis pathways across organisms provides opportunities to adapt mechanisms for short- and medium-chain production to alternative systems. The following section will focus mainly on chain-termination mechanisms, which enable the production of fatty acids with shorter chain lengths than the commonly synthesized palmitic acid (C16:0).

## 5. Chain Termination Mechanisms During Fatty Acid Synthesis

Across organisms, the main mechanism that enables the release of shorter fatty acids from the FAS complex is facilitated by thioesterase enzymes. In rats’ mammary glands, a medium-chain specific thioesterase (thioesterase II) is responsible for the modification of product specificity of FASN to mainly medium-chained (C8–C14) fatty acids. This enzyme is independent and separately encoded from the thioesterase catalytic site of FASN [35]. In other lipogenic tissues of rats, this enzyme is not present, and fatty acid composition consists predominantly of long-chain fatty acids (C16 and longer). On the contrary, in the mammary gland of ruminants, it was shown that the release of fatty acids is independent of thioesterase activity. Moreover, short acyl chain fatty acids are released attached to CoA, while long-chain fatty acids are released from FASN in their free form [25].

In plants and bacteria, the different catalytic sites of FAS exist in the form of monofunctional enzymes, each separately encoded (type II FAS, [19]). Some of these monofunctional enzymes have several variants within the same organism with variable affinities to different lengths of fatty acids (e.g., [36]). Several chain-termination mechanisms were suggested in different plants and were validated in several systems. The main ones that were studied are specific chain length thioesterases and ketoacyl synthase genes (a.k.a condensing enzymes). Heterologous expression of specific thioesterase genes with higher affinity for medium-chain fatty acids increased medium-chain fatty acids accumulation in triglycerides in plants that do not regularly produce these fatty acids [20,37,38,39]. It was shown in several systems that the size of the hydrophobic pocket of the acyl-ACP ketoacyl synthase genes (condensing enzymes) can have an impact on the final chain length [36,40,41]. The effect of this gene alone is relatively weak, but co-expression of medium-chain specific variants together with medium-chain specific thioesterase mounted a synergistic effect [36,40]. The potential of the condensing enzyme to influence the final chain length was also demonstrated in the model yeast. Using targeted mutagenesis in the active sites of the condensing enzyme of FASN resulted in significant amounts of short-chain fatty acids that were released in their free form to the media [42]. Thus, several targets within the fatty acid synthesis pathway have the potential to influence chain length. In most cases, several modifications are needed to gain a significant effect.

## 6. Positional Distribution of Fatty Acids in Triglycerides

In milk fat, fatty acids with specific lengths tend to appear in specific positions on the glycerol backbone of triglycerides. As mentioned, the very short fatty acids (C4:0 and C6:0) are almost exclusively esterified to the sn-3 position in a reaction catalyzed by diacylglycerol *O*-acyltransferase (DGAT). Lauric (C12:0) and Meristic (C14:0) fatty acids are more prominently found in the sn-2 position, and palmitic acid (C16:0) is found mostly in either the sn-1 or sn-2 position [13]. Thus, dairy fat is not just a unique blend of fatty acids, it is also characterized by unique triglycerides with a specific positional distribution. Influencing positional distribution adds another level of complexity to just influencing the chain length of fatty acids. However, some previous studies succeeded in manipulating the positional distribution of specific fatty acids using designated triglyceride synthesis genes. Triglycerides are mainly synthesized through the glycerol 3-phosphate pathway in a multi-step manner. Three different groups of enzymes acylate the three different positions of the glycerol backbone of triglycerides. These include glycerol 3-phosphate acyltransferase (GPAT), lysophosphatidic acid acyltransferase (LPAAT), or 1-acylglycerol-3-phosphate-*O*-acyltransferase (AGPATs) and diacylglycerol *O*-acyltransferase (DGATs). Each of these enzymes is encoded by a group of genes, each with several isoforms within organisms [23]. Unusual fatty acids, produced in organisms that do not normally accumulate them, might not be readily and efficiently utilized into triglycerides. As an example, in rapeseed transformants expressing high levels of heterologous lauryl-ACP thioesterase from California bay, lauric acid was not accumulated in the sn-2 position because of the selectivity of sn-2 acyltransferases against lauric acid. The excess lauric acid was directed to beta-oxidation [43]. Thus, expressing specific triglyceride synthesis genes with varying affinities to the desired fatty acids should be considered to efficiently change positional distribution.

## 7. Milk Lipid Macrostructure—The Milk Fat Globule

Milk fat is secreted in a unique macrostructure termed the milk fat globule (MFG), with a complex structure that, up to date, has not been fully replicated in extra-mammary settings.

The MFG is a lipid–protein assembly that originates from intracellular lipid droplets that are secreted by mammary epithelial cells by the apocrine secretion process [44]. The MFG consists of a core of triglycerides surrounded by a unique tri-layer membrane derived from the endoplasmic reticulum and the plasma membrane of the mammary epithelial cells [45,46]. This outer membrane, known as the milk fat globule membrane (MFGM), is rich in proteins, phospholipids, and glycoproteins [46]. The MFGM was originally considered as an emulsifier of milk fat; however, over the last decade, there has been a growing body of evidence supporting the functional role of the MFGM as a prebiotic milk component [47,48], which was also found to contribute to the immune system development in infants (Reviewed by [49]) and proinflammatory properties in obese adults [50]. The MFGM also plays a role in the digestion and absorption of lipids, carrying bioactive molecules that contribute to the health benefits of milk, including antimicrobial and anti-inflammatory properties [51].

MFGs are the secreted form of intracellular lipid droplets, which are also found in other cell types of mammals and other organisms. The intracellular lipid droplets are stabilized by different proteins and phospholipids, which can vary between organisms and tissues. For example, oleosomes are lipid droplets found in plant seeds, surrounded by a phospholipid monolayer and stabilized by oleosin proteins [52]. These can be extracted intact, leveraging the stability and emulsifying properties of oleosomes to produce plant-based milk alternatives [53]. While technological properties can be maintained, the unique health benefits and specific attributes of MFGs might not be fully replicated. The bioactive components and the complex lipid profile of MFGM are difficult to match, potentially impacting the nutritional quality of the final product [54]. Therefore, while extraction of lipid droplets from plants or microorganisms offers some of the MFG properties, they currently cannot completely substitute the intricate structure and benefits of MFGs.

## 8. Use of Oleaginous Microorganisms for Production of Lipids in Fermentations Systems

Oleaginous microorganisms can include microalgae, yeast, filamentous fungi, and bacteria and are promising candidates for producing lipids by fermentation for various sources [5]. Microbial oil or single-cell oils that are produced by these microorganisms are considered a more sustainable oil source compared to plant-derived oils, and even more so compared to animal-derived fats [55]. Moreover, they can produce oils year-round, independent of climate and geographical location. Various species and strains within this vast category each produce high amounts of lipids, usually in the form of triglycerides with varying fatty acid compositions. Based on their lipid composition, different microorganisms can be used for production of oils for variable applications [5]. The growing interest in sustainable alternatives to dairy fat has focused attention on the potential of oleaginous microorganisms to produce tailored lipids that mimic the unique characteristics of dairy fat.

Microalgae are being in the focus of research because of their natural ability to produce and accumulate essential long-chain polyunsaturated fatty acids [56,57,58]. Therefore, lipids produced by microalgae can be used as food additives and supplements to replace fish-oil-derived essential fatty acids, primarily eicosapentaenoic and docosahexaenoic fatty acids (EPA and DHA, respectively). The production of these essential fatty acids requires a set of specialized desaturases that are either naturally expressed in these strains or were introduced from heterologous sources [59]. However, these fatty acids are found in low amounts in animal fats and especially in dairy fat. Moreover, high concentrations of these long, highly unsaturated fatty acids can negatively impact the technological properties of dairy alternatives like solid fat content.

While microalgae are well-suited for polyunsaturated fatty acids production, other oleaginous microorganisms, such as bacteria, yeast, and yeast-like fungi, offer greater potential for producing saturated and medium-chain fatty acids, which are more characteristic of dairy fat. These types of microorganisms are also at the focus of current research since the higher abundance of saturated fatty acids is also favored for biodiesel production [60,61,62]. Furthermore, advanced genetic engineering tools are available for many oleaginous microorganisms, enabling the production of tailor-made lipids by genetic manipulations. Gene manipulations can be used for both increasing lipid content as well as altering lipid composition. For example, overexpression of an endogenous diacylglycerol O-acyltransferase, DGA1, and acetyl-CoA carboxylase, ACC1, in *Yarrowia lipolytica* resulted in a significant increase in lipid production and content [63]. Approaches to produce medium-chain fatty acids in microorganisms have been mainly focused on heterologous expression of short and medium-chain specific acyl-ACP thioesterase genes with high success in the production of these unusual fatty acids in several systems such as microalgae [37,39] and bacteria [64]. Therefore, although the native lipid composition of oleaginous microorganisms is far from comparable to that of milk fat, using genetic engineering, we can alter gene expression of native genes or introduce heterologous genes such as medium-chain specific thioesterases to shift the production of fatty acids to resemble milk fat.

## 9. Summary and Conclusions

The idea to use oil from oleaginous microorganisms to replace milk fat is relatively innovative, and currently, there are no satisfactory solutions, mainly due to the high complexity of milk lipid composition and structure. Oleaginous microorganisms are promising and sustainable platforms for milk-like lipid production in terms of their ability to accumulate large amounts of triglycerides, utilizing relatively cheap sugar sources and producing year-round in fermenters independently of external conditions. In addition, gene manipulation strategies, which are available for many of these organisms, create the opportunity to alter fatty acid composition in any desired direction. Although there is limited success in incorporating microorganism oil into animal food alternatives, choosing hypothesis-driven genetic targets will enhance our ability to improve dairy alternatives.

## Figures and Tables

**Table 1:** Short- and medium-chain fatty acids in non-ruminant organisms.

Organism Category	Organism Type	Major < 16 Fatty Acids	Source	Storage Form	References
Bacteria	Intestinal bacteria	C2–C4	Fermentation of dietary fiber	Free fatty acids	[26,27]
Plants	Tropical crops (e.g., Coconut and palm)	C8–C14	Seeds	Triglycerides	[28,29]
	Warm climate plants (e.g., *cuphea* spp.)	C8–C12	Seeds	Triglycerides	[30,31]
Animals	Aquatic birds	C12–C14	Preen glands	Wax esters	[32]
	Rodents	C8–C14	Mammary glands	Triglycerides	[33]
	Camelids	C14	Mammary glands	Triglycerides	[34]

## Data Availability

No new data were created or analyzed in this study. Data sharing is not applicable to this article.
